# Towards ultimate parton distributions at the high-luminosity LHC

**DOI:** 10.1140/epjc/s10052-018-6448-y

**Published:** 2018-11-22

**Authors:** Rabah Abdul Khalek, Shaun Bailey, Jun Gao, Lucian Harland-Lang, Juan Rojo

**Affiliations:** 1 Department of Physics and Astronomy, Vrije Universiteit Amsterdam, 1081 HV Amsterdam, The Netherlands; 2 Rudolf Peierls Centre for Theoretical Physics, University of Oxford, Clarendon Laboratory, Parks Road, Oxford, OX1 3PU UK; 3 Institute of Nuclear and Particle Physics, Shanghai Key Laboratory for Particle Physics and Cosmology, School of Physics and Astronomy, Shanghai Jiao Tong University, Shanghai, China; 4 Nikhef Theory Group, Science Park 105, 1098 XG Amsterdam, The Netherlands

## Abstract

Since its start of data taking, the LHC has provided an impressive wealth of information on the quark and gluon structure of the proton. Indeed, modern global analyses of parton distribution functions (PDFs) include a wide range of LHC measurements of processes such as the production of jets, electroweak gauge bosons, and top quark pairs. In this work, we assess the ultimate constraining power of LHC data on the PDFs that can be expected from the complete dataset, in particular after the High-Luminosity (HL) phase, starting in around 2025. The huge statistics of the HL-LHC, delivering ${\mathcal {L}}=3\hbox { ab}^{-1}$ to ATLAS and CMS and ${\mathcal {L}}=0.3\hbox { ab}^{-1}$ to LHCb, will lead to an extension of the kinematic coverage of PDF-sensitive measurements as well as to an improvement in their statistical and systematic uncertainties. Here we generate HL-LHC pseudo-data for different projections of the experimental uncertainties, and then quantify the resulting constraints on the PDF4LHC15 set by means of the Hessian profiling method. We find that HL-LHC measurements can reduce PDF uncertainties by up to a factor of 2 to 4 in comparison to state-of-the-art fits, leading to few-percent uncertainties for important observables such as the Higgs boson transverse momentum distribution via gluon-fusion. Our results illustrate the significant improvement in the precision of PDF fits achievable from hadron collider data alone, and motivate the continuation of the ongoing successful program of PDF-sensitive measurements by the LHC collaborations.

## Introduction

A detailed understanding of the quark and gluon structure of the proton [1–3] is an essential ingredient of theoretical predictions for hadron colliders such as the LHC. This is quantified by the parton distribution functions (PDFs), which determine how the proton’s momentum is shared among its constituents in a hard–scattering collision. PDF uncertainties represent one of the dominant theoretical systematic errors in many important LHC processes, including the profiling of the Higgs boson sector [4]; direct searches for new heavy beyond the Standard Model (BSM) particles [5]; indirect BSM searches by means of the SM Effective Field Theory (SMEFT) [6]; as well as in the measurement of fundamental SM parameters such as the *W* boson mass [7], the Weinberg mixing angle [8], and the strong coupling constant [9] and its running [10].

Since the start of data taking in 2009, the LHC has provided an impressive wealth of information on the proton’s PDFs. Indeed, modern global PDF fits [11–14] include a wide range of LHC measurements in processes such as the production of jets, weak gauge bosons, and top quark pairs. Crucially, the recent breakthroughs in the calculation of NNLO QCD and NLO QED and electroweak corrections (including photon–induced ones) to most PDF–sensitive processes have been instrumental in allowing for the full exploitation of the information provided by the LHC measurements. The impact of high precision LHC data combined with state–of–the art perturbative calculations has been quantified for many of the processes of interest, such as top quark pair production [15, 16], the transverse momentum spectrum of *Z* bosons [17], direct photon production [18, 19], *D* meson production in the forward region [20, 21], *W* production in association with charm quarks [22–24], and inclusive jet production [25, 26]. See the reviews [1, 2] for a more extensive list of references.

With experimentalists warming up to analyse the complete Run II dataset, the high energy physics community is already busy looking ahead to the future. Following Run III, around 2023, a major upgrade of the LHC accelerator and detector systems will make the start of its High Luminosity (HL) operation phase possible. The ten–fold increase in its instantaneous luminosity will lead to the collection of huge datasets, with the HL–LHC expected to deliver around ${\mathcal {L}}=3\hbox { ab}^{-1}$ to ATLAS and CMS and around ${\mathcal {L}}=0.3\hbox { ab}^{-1}$ to LHCb. This unprecedented dataset will open new exciting physics opportunities, such as the measurement of the Higgs boson couplings to second generation fermions as well as of its self–interactions. These opportunities will be summarised in a CERN Yellow Report [27] to be presented before the end of 2018, in order to contribute to the update of the European Strategy for Particle Physics (ESPP).^1^

From the point of view of PDF determinations, the availability of these immense data samples will permit a significant extension of the kinematic coverage of PDF–sensitive measurements as well as a marked improvement in their statistical and systematic uncertainties. With this motivation, the goal of this work is to quantify the impact of the future HL–LHC measurements on the proton PDFs. In other words, we aim to assess the ultimate constraining power of hadron collider data on the PDFs. In turn, the resulting projections for the expected PDF uncertainties will feed into other related projections for HL–LHC processes, which will benefit from the associated reduction of theoretical errors.

It is important to emphasise here that while this type of study has previously been carried out in the context of lepton–hadron colliders such as the Large Hadron electron Collider (LHeC) and the Electron Ion Collider (EIC) [29–37], this is the first time that such a systematic effort has been devoted to determine the PDF–constraining potential of a future hadron collider. Clearly, being able to compare the information on PDFs that will be provided by the HL–LHC with that from proposed electron–proton colliders such as the LHeC represents an important input to inform the upcoming ESPP update.

Our analysis has been carried out as follows. First, we have generated HL–LHC pseudo–data for a number of PDF–sensitive processes: Drell–Yan production (both at high dilepton invariant mass and in the forward rapidity regions); *W* production in association with charm quarks (central and forward regions); inclusive jet and prompt photon production; the transverse momentum of *Z* bosons; and differential distributions in top quark pair production. We have selected those processes that should benefit more directly from the increased statistics available at the HL–LHC. We consider measurements such as inclusive *W*, *Z* production in the central region, which are already completely limited by systematic uncertainties [38, 39], with no significant improvement anticipated from increased statistics alone. For each process, the binning and kinematic cuts applied to the pseudo–data is constructed from a suitable extension of reference measurements at $\sqrt{s}=8$ and 13 TeV. We consider different scenarios for the expected systematic uncertainties, from a conservative one with approximately the same systematics as the corresponding baseline measurements from Run I and a factor of 2 reduction for those from Run II, to an optimistic one with a reduction by a factor 2.5 (5) as compared to Run I (II).

Subsequently, we quantify the constraints of the HL–LHC pseudo–data on the PDF4LHC15_100 set [40–43] by means of the Hessian Profiling method [44] (see also [45]). We have chosen the PDF4LHC15 set since it broadly represents the state–of–the–art understanding of the proton structure. While it is beyond the scope of this work to construct forecasts of the experimental correlation models, we account for their effective impact by using available Run I and II measurements as benchmarks. The resulting profiled sets are then implemented in the LHAPDF6 interface [46], thus being readily available for phenomenological applications.

**Fig. 1:**
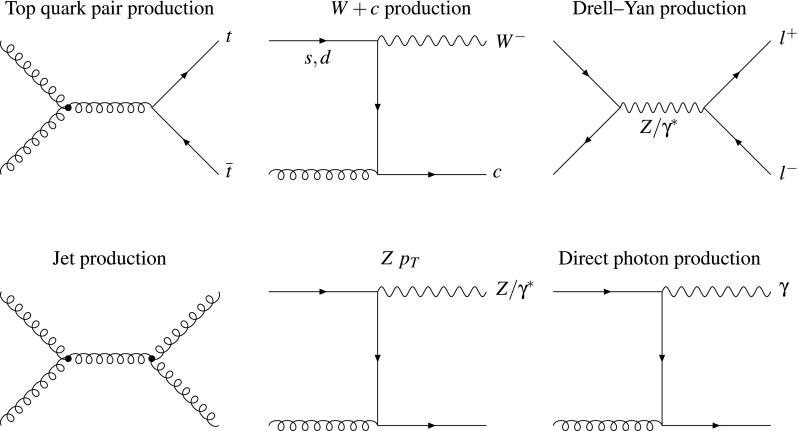
Representative Feynman diagrams at the Born level of the six types of collider processes for which HL–LHC pseudo–data has been generated in this analysis: the production of top quark pairs, *W* bosons in association with charm quarks, and the neutral and charged current Drell–Yan processes; the production of inclusive jets, *Z* bosons at finite transverse momentum, and direct photons

By performing this analysis, we find that the legacy HL–LHC measurements can reduce the uncertainties in the PDF luminosities by a factor between 2 and 5 in comparison to state–of–the–art fits, depending on the specific flavour combination of the initial state and the invariant mass of the produced final state. We also show that our projections for the PDF error reduction, which are predominantly driven by the increased statistics of the HL–LHC data sample, depend only moderately on the specific scenario adopted for the reduction of the experimental systematic errors.

We then explore the implications of the profiled PDFs for representative LHC cross sections at $\sqrt{s}=14$ TeV, both within the Standard Model (SM) and beyond it. Our analysis highlights how ${\mathcal {O}}\left( 1\%\right)$ PDF uncertainties are within the reach of the HL–LHC for key observables such as the transverse momentum distribution in Higgs production from gluon fusion. Therefore, our study illustrates the significant improvement in the precision of PDF determinations achievable from hadron collider data alone, and motivates the continuation of the ongoing successful program of PDF–sensitive measurements at the LHC.

The outline of the paper is the following. First, in Sect. 2 we describe the features of the PDF–sensitive processes used to generate the HL–LHC pseudo–data. Then in Sect. 3 we quantify the constraints on the PDFs of individual processes using the Hessian profiling method. The full set of HL–LHC pseudo–data is combined in Sect. 4 to construct the ultimate HL–LHC parton distributions, which is then used to assess their phenomenological implications for different processes both in the SM and beyond it. Finally, in Sect. 5 we summarise our results and indicate how they are made publicly available.

## Pseudo–data generation

In this section we present the PDF–sensitive processes for which HL–LHC pseudo–data have been generated, provide details about the binning and kinematic cuts, and also describe the baseline Run I and II measurements that are used to model the experimental systematic uncertainties expected in the HL–LHC era.

### PDF–sensitive processes

We start by describing the PDF–sensitive processes that will be considered in this study to generate HL–LHC pseudo–data. Our analysis is based on six different types of processes: the production of top quark pairs, jets, direct photons, and *W* bosons in association with charm quarks, the transverse momentum of *Z* bosons, and the neutral and charged current Drell–Yan processes. In Fig. 1 we show representative Feynman diagrams at the Born level for all of these processes, in order to illustrate their sensitivity to the different partonic initial states. For instance, we see that jets, photon, and top quark pair production are directly dependent on the gluon content of the proton, while *W*+charm is sensitive to strangeness, and the Drell–Yan process to the quark–antiquark luminosity.

This choice of input processes is driven by the fact that some types of hard–scattering reactions should benefit more directly from the increased statistics offered by the HL–LHC than others. Indeed, some of the existing LHC measurements, such as inclusive *W*, *Z* production in the central region [38, 39], are already limited by systematic uncertainties, and therefore are unlikely to improve significantly at higher luminosities. On the other hand, our selection of processes will greatly benefit from the huge HL–LHC dataset either because they are relatively rare, such as *W*+charm, or because their kinematic coverage can be extended to regions of large invariant masses and transverse momentum or forward rapidities where event rates exhibit a steep fall–off. While these pseudo–data sets do include some regions which are currently systematics dominated, i.e. towards central rapidity and lower mass/transverse momentum, as we will see the dominant PDF impact comes from the regions which are not, where the existing data are less constraining and the contributing PDFs are currently less well determined.

In more detail, the specific processes for which HL–LHC pseudo–data have been generated are the following:

- High–mass Drell–Yan, specifically the dilepton invariant mass differential distribution $d\sigma (pp\rightarrow ll)/dm_{ll}$ for $m_{ll}\gtrsim 110$ GeV for a central rapidity acceptance, $|\eta _{l}|\le 2.4$. This process is particularly useful for quark flavour separation, specifically to constrain the poorly known large–*x* sea quarks. Here the ATLAS 8 TeV measurement of differential Drell–Yan cross sections [47] is taken as reference, with additional bins in the high $m_{ll}$ region included to benefit from the enhanced kinematic coverage.
- The differential distributions for on–peak *W* and *Z* boson production in the forward region, $2.0 \le \eta _{l} \le 4.5$, covered by the LHCb experiment. These measurements constrain quark flavour separation, including the strange and charm content of the proton, in the large and small *x* region [48], complementary to the data from the central region. The reference analysis is the LHCb measurement of the rapidity distributions of *W* and *Z* bosons in the muon final state at 8 TeV [49]. In comparison to the reference measurement, a finer binning by a factor of 2 to 5 has been adopted as allowed by the increased event rates. Events are selected if $p_T^l \ge 20$ GeV, the lepton rapidities fall in the LHCb acceptance, and, in the case of *Z* production, there is the additional requirement that $60~\mathrm{GeV}\le m_{ll} \le 120~\mathrm{GeV}$.
- Differential distributions in top quark pair production, providing direct information on the large *x* gluon [15]. Specifically, we consider the top quark transverse momentum $p_T^t$ and rapidity $y_t$, and the top quark pair rapidity $y_{t\bar{t}}$ and invariant mass $m_{t\bar{t}}$. The reference measurements here are the ATLAS 8 TeV differential distributions in the lepton+jets final state [50]. We assume that the statistical correlations between different distributions will be available, as is the case for the 8 TeV data [51], and therefore include the four distributions simultaneously in the fit. To account for the increased statistics of the HL–LHC, the number of bins in the rapidity distributions is doubled, while the $p_T^t$ and $m_{t\bar{t}}$ distributions are extended to higher values in the TeV region.
- The transverse momentum distribution of the *Z* bosons in the dilepton final state, $20 \,\mathrm{GeV}<p_T^{ll}<3.5\,\mathrm{TeV}$ region for central rapidities $|\eta _{Z}|\le 2.4$ and different bins of the dilepton invariant mass $m_{ll}$. This process is relevant to constrain the gluon and the antiquarks at intermediate values of *x* [17]. For the reference analysis, we take the ATLAS measurements of the transverse momentum of lepton pairs at 8 TeV [52]. The pseudo–data is generated for six different bins of the dilepton invariant mass $m_{ll}$, with boundaries 12, 20, 30, 40, 66, 116, and 150 GeV respectively. In each of the invariant mass $m_{ll}$ bins, additional bins are added to the $p_T^{ll}$ distribution to exploit the improved coverage of the large transverse momentum region.
- The production of *W* bosons in association with charm quarks. This process provides a sensitive handle on the strangeness content of the proton [23, 53], which is the least well known of the light quark PDFs. The pseudo–data for this process has been generated as a function of the lepton psuedorapidity $\eta _l$ from the *W* boson decay, and is inclusive over the kinematics of the charm quark provided it satisfies the selection cuts. For this process, pseudo–data have been generated both for the central rapidity region relevant for ATLAS and CMS as well as for the forward region covered by LHCb. In the central rapidity region, $|\eta ^l|\le 2.4$, the reference measurement is the CMS analysis at 13 TeV [24], where events are selected provided that $p_T^c \ge 5$ GeV and $p_T^l \ge 26$ GeV with *l* indicating the result of the $W\rightarrow l\nu$ decay. At forward rapidities, $2\le \eta ^l \le 4.5$, we use a dedicated selection strategy with $2.2 \le \eta ^c \le 4.2$, $p_T^\mu \ge 20$ GeV, $p_T^c \ge 20$ GeV, and $p_T^{\mu +c}\ge 20$ GeV [54]. We take the acceptance to be 30% due to *c*–jet tagging and an overall normalisation error of 5%.
- Prompt isolated photon production represents a complementary probe of the gluon PDF at intermediate values of *x* [19]. Here the pseudo–data has been generated as differential distributions in the photon transverse momentum $p_T^\gamma$ for different bins in the photon rapidity $\eta ^\gamma$. The reference measurements here is the ATLAS 13 TeV analysis of [55], where additional bins have been added to the $p_T^\gamma$ distribution in each rapidity bins to benefit from the improved coverage of the large $p_T^\gamma$ region.
- Finally, we consider the inclusive production of hadronic jets in different bins of their rapidity up to $|y_\mathrm{jet}|\le 3$ as a function of their $p_T^{\mathrm{jet}}$. This process provides direct information on the gluon and the valence quarks at large–*x*. Here the jets are reconstructed using the anti–$k_T$ algorithm with $R=0.4$ as radius parameter. The reference measurement here is the 13 TeV ATLAS analysis of inclusive jet and dijet production based on a luminosity ${\mathcal {L}}=3.2$ $\hbox {fb}^{-1}$ from the 2015 data–taking period. The coverage of the high–$p_T$ region has been extended to the $p_T^{\mathrm{jet}}\simeq 2-3$ TeV in comparison to these.

It is important to emphasise that the list of processes considered in this work is by no means exhaustive. Clearly, there are other important processes that will provide useful information on the proton PDF in the HL–LHC era. Among these, one could consider dijet production [56] and single top quark production [57], providing information on the gluon and on the quark flavour separation respectively. In both cases, the NNLO corrections for differential distributions are available, as well as reference LHC measurements at 8 and 13 TeV. Our choices of processes are in addition generally geared towards the high and intermediate *x* region. Other PDF–sensitive processes, such as inclusive *D* meson production, can play a role at lower *x*. Although in this case it is unlikely to benefit from the high–luminosity phase, as it already occurs at very high rates, this may not be true for other rarer processes sensitive to this region.

In addition, one should take into account that progress from both the experimental and theoretical sides could lead to novel processes being added to the PDF fitting toolbox, for instance more exclusive processes or processes for which the standard DGLAP description breaks down. With these caveats, the set of processes adopted in this work is representative enough to provide a reasonable snapshot of the PDF–constraining potential of the HL–LHC.

It is also important to mention that the HL–LHC projections presented in this work are based on pseudo–data generated specifically for this study, and that they are not endorsed by the LHC collaborations. However, we have taken into account all the feedback and suggestions received from the ATLAS, CMS, and LHCb contacts involved in the Yellow Report studies.

### Theory calculations and pseudo–data generation

For the various processes described above, we have generated pseudo–data for a centre–of–mass energy of $\sqrt{s}=14$ TeV assuming a total integrated luminosity of ${\mathcal {L}}=3$ $\hbox {ab}^{-1}$ for the CMS and ATLAS experiments, and of ${\mathcal {L}}=0.3\hbox { ab}^{-1}$ for the LHCb experiment. Note that in the former case we explicitly include pseudo–data from both experiments. Statistical uncertainties are evaluated from the expected number of events per bin, taking into account branching ratios and acceptance corrections determined from the corresponding reference analysis. Systematic uncertainties are taken to be those of the 13 or 8 TeV baseline analyses and then rescaled appropriately. We consider various scenarios for the reduction of systematic errors, from a more conservative one to a more optimistic one.

**Table 1:** Summary of the features of the HL–LHC pseudo–data generated for the present study For each process we indicate the kinematic coverage, the number of pseudo–data points used across all detectors $N_\mathrm{dat}$, the values of the correction factors $f_\mathrm{corr}$ and $f_\mathrm{red}$; and finally the reference from the 8 TeV or 13 TeV measurement used as baseline to define the binning and the systematic uncertainties of the HL–LHC pseudo–data, as discussed in the text

Process	Kinematics	$N_\mathrm{dat}$	$f_\mathrm{corr}$	$f_\mathrm{red}$	Baseline
$Z\,p_T$	$20\,\mathrm{GeV}\le p_T^{ll} \le 3.5$ TeV	338	0.5	$\left( 0.4, 1\right)$	[52] (8 TeV)
	$12\,\mathrm{GeV}\le m_{ll} \le 150$ GeV				
	$$|y_{ll}|\le 2.4$$				
High-mass Drell-Yan	$p_T^{l1(2)}\ge 40(30)\,\mathrm{GeV}$	32	0.5	$\left( 0.4, 1\right)$	[47] (8 TeV)
	$|\eta ^l|\le 2.5$, $m_{ll}\ge 116\,\mathrm{GeV}$				
Top quark pair	$m_{t\bar{t}}\simeq 5$ TeV, $|y_t|\le 2.5$	110	0.5	$\left( 0.4, 1\right)$	[50] (8 TeV)
*W*+charm (central)	$p_T^\mu \ge 26\,\mathrm{GeV}$, $p_T^c \ge 5\,\mathrm{GeV}$	12	0.5	$\left( 0.2, 0.5\right)$	[24] (13 TeV)
	$$|\eta ^\mu |\le 2.4$$				
*W*+charm (forward)	$p_T^\mu \ge 20\,\mathrm{GeV}$, $p_T^c \ge 20\,\mathrm{GeV}$	10	0.5	$\left( 0.4, 1\right)$	LHCb projection
	$$p_T^{\mu +c} \ge 20\,\mathrm{GeV}$$				
	$2\le \eta ^\mu \le 4.5$, $2.2\le \eta ^c \le 4.2$				
Direct photon	$E_T^\gamma \lesssim 3$ TeV, $|\eta _{\gamma }|\le 2.5$	118	0.5	$\left( 0.2, 0.5\right)$	[55] (13 TeV)
Forward *W*, *Z*	$p_T^{l}\ge 20\,\mathrm{GeV}$, $2.0\le \eta ^l\le 4.5$	90	0.5	$\left( 0.4, 1\right)$	[49] (8 TeV)
	$$60\,\mathrm{GeV}\le m_{ll}\le 120\,\mathrm{GeV}$$				
Inclusive jets	$|y| \le 3$, $R = 0.4$	58	0.5	$\left( 0.2, 0.5\right)$	[61] (13 TeV)
Total		768			

Theoretical predictions are computed at next–to–leading order (NLO) in the QCD expansion using MCFM [58] interfaced to APPLgrid [59] to produce the corresponding fast grids. The only exception is inclusive jet production, for which the NLO calculation is obtained from the NLOJET++ program [60]. The central value of the pseudo–data initially coincides with the corresponding prediction obtained using this NLO calculation with the PDF4LHC15 NNLO set as input. Subsequently, this central value is fluctuated according to the corresponding experimental uncertainties. This implies that, by construction, one should find $\chi ^2/N_\mathrm{dat}\simeq 1$ from the fit to the pseudo–data.

Specifically, if $\sigma _i^{\mathrm{th}}$ is the theoretical cross section for bin *i* of a given process, then the central value of the HL–LHC pseudo–data $\sigma _i^{\mathrm{exp}}$ is constructed by means of

2.1 $$\begin{aligned} \sigma _i^{\mathrm{exp}} = \sigma _i^{\mathrm{th}} \times \left( 1 + r_i\cdot \delta ^{\mathrm{exp}}_{\mathrm{tot},i} + \lambda \cdot \delta ^{\mathrm{exp}}_{{\mathcal {L}}}+ s\cdot \delta ^{\mathrm{exp}}_{{\mathcal {N}}}\right) , \end{aligned}$$

where $r_i$, $\lambda$, and *s* are univariate Gaussian random numbers, $\delta ^\mathrm{exp}_{\mathrm{tot},i}$ is the total (relative) experimental uncertainty corresponding to this specific bin (excluding the luminosity and normalization uncertainties), and $\delta ^{\mathrm{exp}}_{{\mathcal {L}}}$ is the luminosity uncertainty, which is fully correlated among all the pseudo–data bins of the same experiment (but uncorrelated among different experiments). We take this luminosity uncertainty to be $\delta ^{\mathrm{exp}}_{{\mathcal {L}}}=1.5\%$ for the three LHC experiments. $\delta ^{\mathrm{exp}}_{{\mathcal {N}}}$ are possible additional normalization uncertainties as in the case of *W* boson production in association with charm quarks that will be explained later.

In Eq. (2.1), the total experimental uncertainty $\delta ^{\mathrm{exp}}_{\mathrm{tot},i}$ is defined as

2.2 $$\begin{aligned} \delta ^{\mathrm{exp}}_{\mathrm{tot},i} \equiv \left( \left( \delta ^{\mathrm{exp}}_{\mathrm{stat},i}\right) ^2 + \left( f_\mathrm{corr}\times f_\mathrm{red}\times \delta ^{\mathrm{exp}}_{\mathrm{sys},i}\right) ^2 \right) ^{1/2}. \end{aligned}$$

In this expression, the relative statistical error $\delta ^\mathrm{exp}_{\mathrm{stat},i}$ is computed as

2.3 $$\begin{aligned} \delta ^{\mathrm{exp}}_{\mathrm{stat},i} = \left( f_\mathrm{acc} \times N_{\mathrm{ev},i}\right) ^{-1/2}, \end{aligned}$$

where $N_{\mathrm{ev},i}=\sigma _i^{\mathrm{th}} \times {\mathcal {L}}$ is the expected number of events in bin *i* at the HL–LHC with ${\mathcal {L}}=3~(0.3)$ $\hbox {ab}^{-1}$. In Eq. (2.3), $f_\mathrm{acc}\le 1$ is an acceptance correction which accounts for the fact that, for some of the processes considered, such as top quark pair production, there is a finite experimental acceptance for the final state products and/or one needs to include the effects of branching fractions. The value of $f_\mathrm{acc}$ is determined by extrapolation using the reference dataset, except for forward *W*+charm production (where there is no baseline measurement) where the acceptance is set to $f_\mathrm{acc}=0.3$, due dominantly to the *c*–jet tagging efficiency.

In Eq. (2.2), $\delta ^{\mathrm{exp}}_{\mathrm{sys},i}$ indicates the total systematic error of bin *i* taken from the reference LHC measurement at either 8 TeV or 13 TeV, while $f_\mathrm{red}\le 1$ is a correction factor that accounts for the fact that on average systematic uncertainties will decrease at the HL–LHC in comparison to Run II due to both detector improvements and the enlarged dataset for calibration. Finally, $f_\mathrm{corr}$ represents an effective correction factor that accounts for the fact that data with correlated systematics may be more constraining than the same data where each source of error is simply added in quadrature, as we do in this analysis. We discuss below in Sect. 2.3 how the value of $f_\mathrm{corr}$ can be determined by means of available LHC measurements for which the full information on correlated systematics is available.

Concerning the theoretical calculations adopted here, since the present study relies on pseudo–data, it is not necessary to account for higher–order QCD effects or electroweak corrections. Indeed, by far the dominant contribution to the PDF sensitivity of hadron collider processes is contained within the NLO calculation. As in the case of PDF closure tests [62], here we are only interested in the relative reduction of the PDF uncertainties once the HL–LHC data is included in the fit, while the central value itself will be essentially unaffected. Note that this also holds for the contribution of photon–initiated (PI) processes, since the photon PDF is very well know [63–65]. Therefore, PI processes effectively induce an overall rescaling of the cross section which becomes irrelevant when generating pseudo–data.

In Table 1 we present the summary of the main features of the HL–LHC pseudo–data generated for the present study. For each process, we indicate the kinematic coverage, the number of pseudo–data points used $N_\mathrm{dat}$, the values of the correction factors $f_\mathrm{acc}$, $f_\mathrm{corr}$, and $f_\mathrm{red}$; and finally the reference for the 8 TeV or 13 TeV measurement used as baseline to define the binning and the systematic uncertainties of the HL–LHC pseudo–data. A total of around $N_\mathrm{dat}= 768$ pseudo–data points are then used in the PDF profiling. The values of the reduction factor for the systematic errors $f_\mathrm{red}$ is varied between 1 (0.5) and 0.4 (0.2) in the conservative and optimistic scenarios for a 8 TeV (13 TeV) baseline measurement. This different treatment is motivated by the fact that available 13 TeV measurements are based on a smaller dataset and therefore tend to have larger systematic errors in comparison to the 8 TeV case. Thus we can expect some improvement here at the HL–LHC even in the most conservative scenario; Run II measurements based on the complete integrated luminosity will certainly benefit from reduced systematics.

**Fig. 2:**
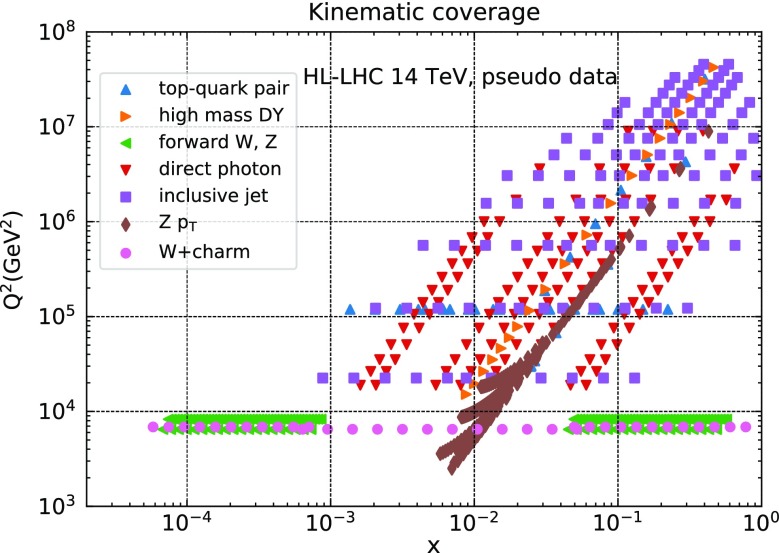
The kinematical coverage in the $(x,Q^2)$ plane of the HL–LHC pseudo–data included in this analysis. For each data point, the values of $(x_1,Q^2)$ and $(x_2,Q^2)$ corresponding to each of the two colliding partons are determined approximatly from the corresponding leading–order kinematics. We assume $x_1=x_2$ if rapidities are not specified for the final states. The HL–LHC pseudo–data therefore spans a wide region in the kinematic plane, namely $6\times 10^{-5} \lesssim x \lesssim 0.7$ and $40~\mathrm{GeV} \lesssim Q \lesssim 7~\mathrm{TeV}$

In Fig. 2 we show the kinematical coverage in the $(x,Q^2)$ plane of the HL–LHC pseudo–data included in this analysis. For each data point, the values of $(x_1,Q)$ and $(x_2,Q)$ corresponding to the two colliding partons are determined approximately from leading–order kinematics, which is sufficient for illustration purposes. We assume $x_1=x_2$ if rapidities are not specified for the final states. We see that the HL–LHC pseudo–data covers a wide kinematic region, including the large momentum transfers up to $Q\simeq 6$ TeV, as well as the large-*x* region, with several different processes. Specifically, the input pseudo–data spans the range $6\times 10^{-5} \lesssim x \lesssim 0.7$ and $40~\mathrm{GeV} \lesssim Q \lesssim 7~\mathrm{TeV}$ in the (*x*, *Q*) kinematic plane. Note that the LHCb measurements are instrumental to constrain the small–*x* region, $6\times 10^{-5} \lesssim x \lesssim 10^{-3}$, beyond the acceptance of ATLAS and CMS.

### Impact of correlating uncertainties

As we will also discuss in Sect. 3, when constructing the $\chi ^2$ estimator for the HL–LHC pseudo–data we will not explicitly include the correlations between the systematic errors. Instead, we add statistical and systematic uncertainties in quadrature as indicated in Eq. (2.2). This choice is motivated by the fact that it is already challenging to estimate how specific systematic uncertainties will be reduced at the HL–LHC, let alone how their mutual correlations will be modified. Note that even restricting ourselves to Run I measurements, the determination of the experimental correlation model is a delicate problem, and can in some cases complicate the PDF interpretation of measurements such as inclusive jet production [66].

On the other hand, completely neglecting the effects of the experimental correlations may artificially reduce the impact of the pseudo–data into the fit. Precisely for this reason, we have introduced the correction factor $f_\mathrm{corr}$ in Eq. (2.2). Its value has been tuned to the LHC measurements of the top quark pair differential distributions [50, 67] at $\sqrt{s}=8$ TeV and of the central $W+$charm rapidity distribution [24] at $\sqrt{s}=13$ TeV, for which the full breakdowns of systematic errors are available. Although not shown explicitly here, in the latter case we also check against the published 7 TeV data [68], finding similar results to the preliminary 13 TeV. In the following comparison, we use a value for the tolerance of $T=1$ (defined in the next section) to exaggerate the effect of $f_\mathrm{corr}$ due to the new data having a more dominate role in the $\chi ^2$, enabling an easier determination of the optimal value.

**Fig. 3:**
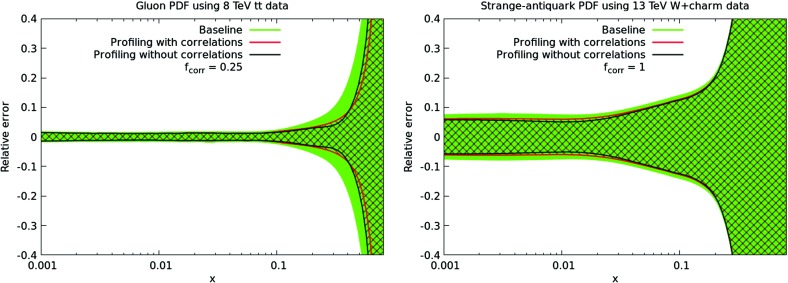
Comparison between the baseline PDF4LHC15 set and the sets profiled with the LHC data, either with or without the correlations between the experimental systematic uncertainties accounted for. In the latter case, the $f_\mathrm{corr}$ factor is chosen to reproduce the results of the profiling when the correlations are included, see text. We show here the results of profiling with the top differential distributions at $\sqrt{s}=8$ TeV with $f_\mathrm{corr}= 0.25$ (left) and the $W+$charm rapidity distribution at $\sqrt{s}=13$ TeV with $f_\mathrm{corr}= 1$ (right plot). A tolerance factor of $T=1$ has been used for this specific comparison

In Fig. 3 we compare the baseline PDF4LHC15 set and the sets profiled with these two LHC datasets, with or without the correlations between the experimental systematic uncertainties accounted for. In the latter case, the $f_\mathrm{corr}$ factor is chosen to reproduce the results of the profiling when the correlations are included. We can see that for the two considered datasets, rather different values of $f_\mathrm{corr}$ are preferred; for the top data, we require $f_\mathrm{corr}\sim 0.25$ while for the $W+$charm data we require instead $f_\mathrm{corr}\sim 1$. Clearly the precise value of this correction therefore appears to depend quite sensitively on the considered datasets, in terms of the corresponding breakdown of systematic uncertainties and overall PDF impact.

The results of Fig. 3 might suggest that, for projections which are dominantly driven by the potential improvement in systematic uncertainties, our approach could be questionable and require a more complete treatment of experimental correlations. However, here we have explicitly chosen our input dataset to be composed of those processes for which the PDF impact will be driven instead by the improvement in the statistics and extension to unconstrained kinematic regions. Indeed, we will see later on that the specific value of this parameter does not have a large impact on the final results, and we will simply take $f_\mathrm{corr}=0.5$ in what follows as an average, somewhat weighted towards the value required by the top quark differential data, as this shows a larger PDF impact and would therefore be more important to account for accurately.

## HL–LHC constraints from individual processes

In this section, we study the constraints on the PDFs that are expected from individual HL–LHC measurements listed in Table 1. First of all, we review the formulation of the Hessian profiling used in this work to quantify the PDF constraints. Then we present the results for the various HL–LHC processes and study how the description of the pseudo–data is affected. The complete set of processes is combined together into a single profiled PDF set in the next section.

### The Hessian profiling method

Quantifying the impact of new experimental data into a Hessian PDF set such as PDF4LHC15_100 can be efficiently carried out by means of the Hessian Profiling technique [44, 45]. This approach is based on the minimization of the following figure of merit:

3.1 $$\begin{aligned}&\chi ^2\left( \mathrm{\beta _{exp}},\mathrm{\beta _{th}}\right) \nonumber \\&\quad =\sum _{i=1}^{N_\mathrm{dat}}\frac{1}{\left( \delta ^{\mathrm{exp}}_{\mathrm{tot},i}\sigma ^{\mathrm{th}}_i\right) ^2}\left( \sigma _i^{\mathrm{exp}} +\sum _j\Gamma _{ij}^{\mathrm{exp}}\beta _{j,\mathrm exp} -\sigma _i^{\mathrm{th}} +\sum _k\Gamma _{ik}^{\mathrm{th}}\,\beta _{k,\mathrm th} \right) ^2 \nonumber \\&\qquad +\sum _j \beta _{j,\mathrm exp}^2+T^2\sum _k \beta _{k,\mathrm th}^2 \; , \end{aligned}$$

where $\sigma _i^\mathrm{exp}~(\sigma _i^{\mathrm{th}})$ are the central values of a given experimental measurement (theory prediction), see Eq. (2.1), $\beta _{j,\mathrm exp}$ are the nuisance parameters corresponding to the set of fully correlated experimental systematic uncertainties, $\beta _{k,\mathrm th}$ are the nuisance parameters corresponding to the PDF Hessian eigenvectors, $N_\mathrm{dat}$ is the number of data points and *T* is the tolerance factor. The matrices $\Gamma _{ij}^{\mathrm{exp}}$ and $\Gamma _{ik}^{\mathrm{th}}$ encode the effects of the corresponding nuisance parameters on the experimental data and on the theory predictions, respectively.

**Fig. 4:**
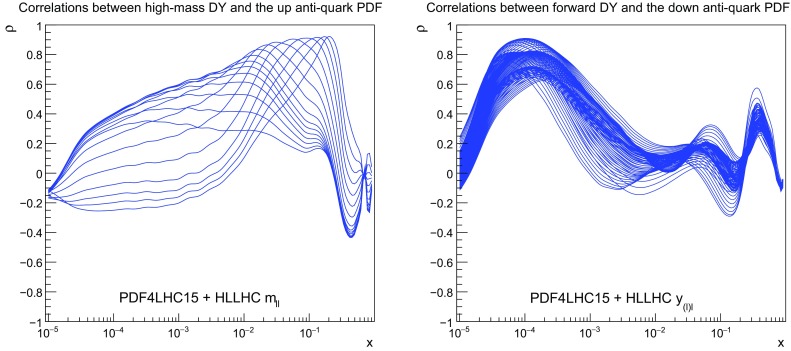
The correlation coefficients $\rho$ between the PDFs and the HL–LHC pseudo–data. Left: the correlation between the anti–up quark and the high–mass Drell–Yan pseudo–data as a function of *x* for $Q=100$ GeV. Right: the correlation between the anti–down quark and the inclusive *W*, *Z* production process in the forward region. In each plot, the different curves correspond to each of the bins of the pseudo–data used in the fit

The minimisation of Eq. (3.1) produces approximately equivalent results to carrying out the corresponding Hessian fit from scratch, provided settings such as the input PDF parameterisations, the tolerance factor *T*, and the theoretical calculations are unchanged. An advantage of the Hessian profiling method in comparison to related techniques such as the Bayesian reweighting method [69, 70], relevant for Monte Carlo PDF sets, is that there is no information loss even when the added measurements provide significant new information. This property is crucial in the present analysis, since the HL–LHC pseudo–data induces significant constraints on the PDFs.

As mentioned in Sect. 2.2, in this study we add in quadrature statistical and experimental uncertainties (except for the luminosity, which is kept fully correlated), and then account for the effects of the missing correlations by means of the factor $f_\mathrm{corr}$. For this reason, we only consider nuisance parameters for the luminosity errors, as well as for an overall normalization uncertainty of 5% in forward *W*+charm production, arising from charm–jet tagging. If we then minimise Eq. (3.1) with respect to these experimental nuisance parameters we obtain

3.2 $$\begin{aligned}&\chi ^2\left( \mathrm{\beta _{th}}\right) =\sum _{i,j=1}^{N_\mathrm{dat}}\left( \sigma _i^{\mathrm{exp}} -\sigma _i^{\mathrm{th}} +\sum _k\Gamma _{ik}^{\mathrm{th}}\,\beta _{k,\mathrm th}\right) \left( \text {cov} \right) _{ij}^{-1}\nonumber \\&\qquad \times \left( \sigma _j^{\mathrm{exp}} -\sigma _j^{\mathrm{th}} +\sum _m\Gamma _{jm}^{\mathrm{th}}\,\beta _{m,\mathrm th}\right) +T^2\sum _k \beta _{k,\mathrm th}^2,\nonumber \\ \end{aligned}$$

where we have defined the experimental covariance matrix as follows:

3.3 $$\begin{aligned} \left( \text {cov} \right) _{ij} = \delta _{ij}\left( \delta ^{\mathrm{exp}}_{\mathrm{tot},i}\sigma ^{\mathrm{th}}_i\right) ^2 \!+\! \Gamma _{i,\text {lumi}}^\mathrm{exp}\Gamma _{j,\text {lumi}}^{\mathrm{exp}} + \Gamma _{i,\text {norm}}^\mathrm{exp}\Gamma _{j,\text {norm}}^{\mathrm{exp}}.\nonumber \\ \end{aligned}$$

Note that since Eq. (3.3) is defined in terms of a fixed theoretical prediction (rather than of the fit output itself), our results are resilient with respect to the D’Agostini bias [71, 72].

At this point, the minimisation of Eq. (3.2) with respect to the Hessian PDF nuisance parameters $\beta _{k,\mathrm th}$ can be interpreted as leading to PDFs that have been optimized to describe this new specific measurement. The resulting Hessian matrix in the $\beta _{k,\mathrm th}$ parameter space at the minimum can be diagonalized to construct the new eigenvector directions, and PDF uncertainties are determined from the $\Delta \chi ^2=T^2$ criteria. In the studies presented here, we use $T=3$, which roughly corresponds to the average tolerance determined dynamically in the CT14 and MMHT14 analyses. The resulting profiled PDF set^2^ can be straightforwardly used for phenomenology using the uncertainty prescription of symmetric Hessian sets, and the default output format is compliant with the LHAPDF interface.

### Inclusive gauge boson production

We now present results for the Hessian profiling of the PDF4LHC15 set after the inclusion of HL–LHC pseudo–data from individual processes. Then in Sect. 4 we will consider the results of the combination for all the processes together. We begin with the inclusive gauge boson production processes listed in Table 1 and described in Sect. 2.1. We consider two sets of pseudo–data: inclusive $\gamma ^*/Z$ production in the central rapidity $|\eta _{ll}|\le 2.4$ and high invariant mass $m_{ll}\ge 116$ GeV regions, and inclusive $W^+,W^-,\gamma ^*/Z$ production in the forward region, $2.0\le \eta ^l\le 4.5$.

**Fig. 5:**
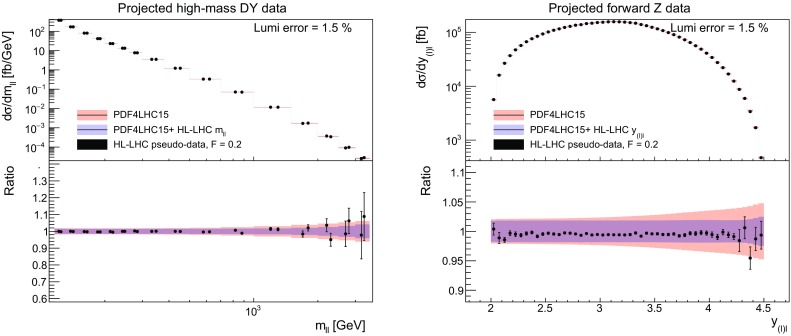
Comparison between the HL–LHC pseudo–data and the theoretical predictions for high–mass (left) and forward (right) Drell–Yan production. The theory calculations are shown both before (PDF4LHC15) and after profiling. Luminosity uncertainties are not shown in the experimental errors. In the bottom panel, we show the same results normalised to the central value of the original theory calculation. Note in the right plot the comparison are only made for forward *Z* data though both *W* and *Z* data are included in the profiling

In this section, we will use the same structure to discuss the impact on the PDFs of the individual HL–LHC processes that are being considered. First, we will display representative examples of the correlations between the PDFs and the pseudo–data, to illustrate the sensitivity of the latter. Second, we will show how the description of the HL–LHC pseudo–data is modified once it is included in the PDF4LHC15 set by means of profiling. Finally, we will assess its impact on the PDFs in a specific scenario for the projections of the experimental systematic errors. In particular, we adopt the ‘optimistic’ choice of Table 2, i.e. $F\equiv f_\mathrm{corr}\cdot f_\mathrm{red}=0.2$, which corresponds to a value $f_\mathrm{red}=0.4$ for the reduction of the systematic uncertainties compared to the 8 TeV baseline measurements. As discussed above, for 13 TeV baselines, in this scenario we take a lower value of $f_\mathrm{red}=0.2$, to account for the smaller 13 TeV datasets these are based on.

We start by discussing the correlations. In Fig. 4 we show the correlation coefficients $\rho$ between the PDFs and the HL–LHC pseudo–data on the Drell–Yan process. The left (right) plot displays the correlation between the anti–up (anti–down) quark as a function of *x* for $Q=100$ GeV for the high–mass (forward) Drell–Yan pseudo–data. A value of $\rho$ close to 1 ($-1$) in a given region of *x* indicates that this process is strongly (anti–) correlated with the input PDFs in this same region, and thus that could potentionally be used to reduce PDF uncertainties there.

As we can see from Fig. 4, in the case of high–mass Drell–Yan we have $\rho \ge 0.9$ for $0.05 \lesssim x \lesssim 0.5$, indicating that this process can provide information on the large–*x* antiquarks. In the case of the forward *W*, *Z* production measurements the correlation coefficient for the $\bar{d}$ PDF peaks at $x\simeq 10^{-4}$, highlighting that the forward kinematic coverage of LHCb allows the quark flavour separation to be pinned down to small values of *x*.

We next assess the impact of inclusive gauge boson production, after profiling. In Fig. 5 we show the comparison between the HL–LHC pseudo–data and the theoretical predictions for high–mass (left) and forward (right) Drell–Yan production. Note in the right plot the comparison is only made for forward *Z* data, but both *W* and *Z* data are included in the profiling. In addition, in the left plot, in each bin there are two experimental pseudo–data points, corresponding to the ATLAS and CMS projections; this is true for all central rapidity pseudo–datasets which follow. The theory calculations are shown both before (that is, using the PDF4LHC15 set) and after profiling. Luminosity uncertainties are not shown in the experimental errors, but are included in the profiling. In the bottom panel, we show the same results normalised to the central value of the original theory calculation.

**Fig. 6:**
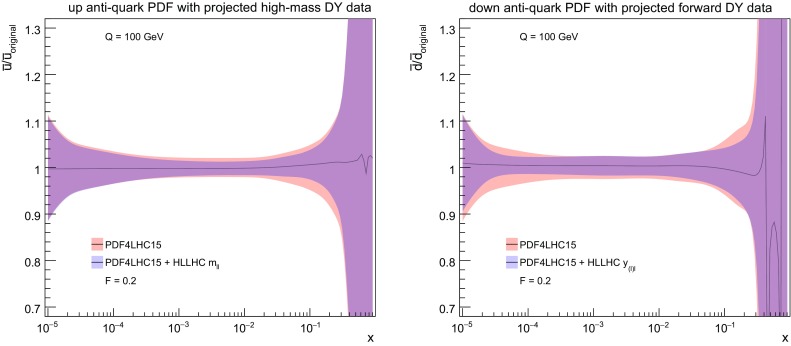
The impact of the HL–LHC pseudo–data on the PDFs at $Q=100$ GeV. Left: impact of high–mass Drell–Yan production on the up antiquark. Right: impact of forward *W*, *Z* process on the down antiquark

From these comparisons, we see that the impact of the high–mass Drell–Yan pseudo–data on the PDFs is rather moderate, presumably because even at the HL–LHC the expected precision of the measurements is comparable or larger than current PDF uncertainties, in particular in the high $m_{ll}$ range. On the other hand, for the *W*, *Z* measurements that will be carried out by LHCb we can observe a marked error reduction of up to a factor two, highlighting the usefulness of the forward kinematic coverage. Note that in both cases the central values of the theoretical predictions are relatively unaffected, with the dominant impact being on the uncertainties. This is expected, as by construction we assume the datasets are consistent with the underlying theory and PDFs.

Concerning the corresponding impact of the HL–LHC pseudo–data on the PDFs, in Fig. 6 we show the reduction of the PDF uncertainties found upon the inclusion of the high–mass Drell–Yan (left) and the forward *W*, *Z* (right) pseudo–data on the PDF4LHC15 set. We display the same PDF flavours as those used in the calculation of the correlation coefficients in Fig. 4, namely the up and down antiquarks respectively. What we find is consistent with Fig. 5: a rather moderate effects on the up antiquark from the high–mass Drell–Yan process, while a more marked effect on the down antiquark from the forward *W*, *Z* process specially in the small–*x* region.

### Top quark pair production

**Fig. 7:**
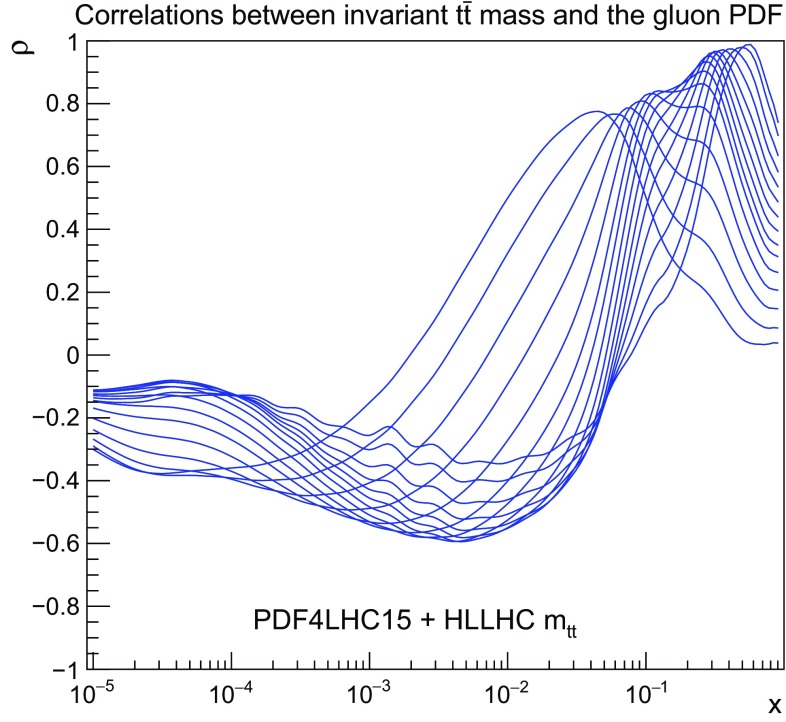
As in Fig. 4, now for the correlation coefficient between the gluon PDF and the various bins of $m_{t\bar{t}}$, the invariant mass of the top quark pair

Here we will focus on the gluon PDF, given that at the LHC top quark pairs are mostly produced via gluon fusion. As explained in Sect. 2.1, we include four different distributions simultaneously: $p_T^t$, $y_t$, $m_{t\bar{t}}$, and $y_{t\bar{t}}$, assuming that the statistical correlations among them will be available. First, in Fig. 7 we show the same correlation coefficients as in Fig. 4 now between the gluon PDF and the various bins of $m_{t\bar{t}}$, the invariant mass distribution of top quark pairs. The fact that $\rho$ peaks in the large–*x* region indicates that adding the $t\bar{t}$ distributions will directly constrain the gluon PDF here.

**Fig. 8:**
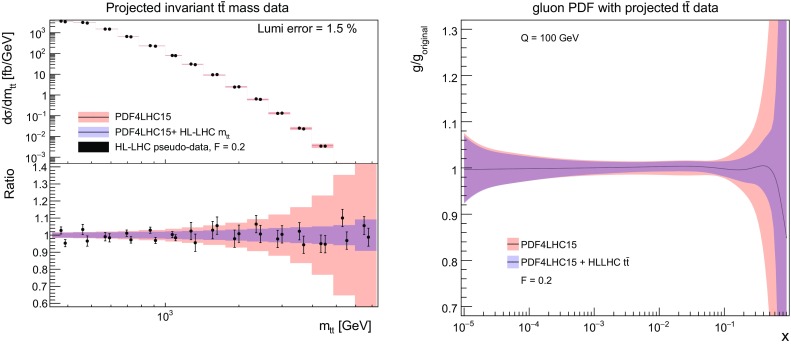
Left: As in Fig. 5, now for the $m_{t\bar{t}}$ distribution in top quark pair production. Right: As in Fig. 6, now for the gluon PDF after including the HL–LHC $t\bar{t}$ pseudo–data in the fit

Next, in Fig. 8 (left) we show the same comparison as in Fig. 5 now for the $m_{t\bar{t}}$ distribution. We can observe a very marked PDF uncertainty reduction at large values of the invariant mass. As expected, we find in Fig. 8 (right) that the addition of the HL–LHC $t\bar{t}$ pseudo–data leads to a significant reduction in the PDF uncertainties in the gluon PDF at large–*x*, highlighting the good constraining power of this type of measurements.

### Jet and photon production

**Fig. 9:**
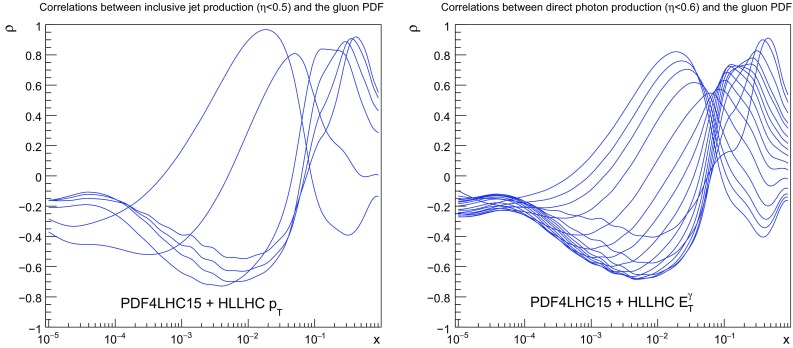
As in Fig. 4, now for the correlation coefficient between the gluon PDF and the central rapidity bin of the inclusive jet (left) and direct photon (right) pseudo–data

We now turn to consider two of the processes that can be used to provide information on the gluon: inclusive jet production and direct photon production. Note that these measurements also provide a handle on the valence quark distributions, due to the significant fraction of events that originate from quark–gluon scattering. In Fig. 9 we display the correlation coefficient between the gluon PDF and the central rapidity bin of the inclusive jet (left) and direct photon (right) pseudo–data. From this comparison we see that the correlation profiles are similar for the two processes. In both cases the correlation coefficient is significant around $x\simeq 10^{-2}$, is then reduced a bit, and then becomes large again, peaking at $x\simeq 0.5$. One can verify that the value of $\rho$ decreases as we move to more forward rapidities, due to the enhanced contribution from quark–initiated diagrams.

**Fig. 10:**
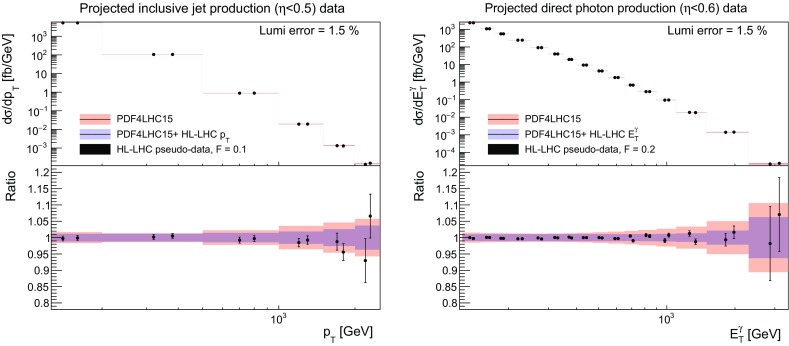
As in Fig. 5, now for the central rapidity bins of the inclusive jet production (left plot) and the direct photon production (right plot) HL–LHC pseudo–data

The corresponding comparison between the theory predictions and the HL–LHC pseudo–data, before and after adding the latter in the fit, is shown in Fig. 10. Note that while we show the results only for central rapidity bins, the PDF fits include the constraints from all the available rapidity bins. It is interesting to observe the excellent coverage that the HL–LHC will offer in the TeV region for these two processes. In the case of inclusive jet (direct photon) production, we expect to measure the differential cross sections up to $p_T^{\mathrm{jet}}\simeq 2-3$ TeV ($E_T^\gamma \simeq 3$ TeV), a marked improvement in comparison to current coverage. Given the back–to–back topology at Born level for these two processes, we see that they can probe scales up to $Q\simeq 6$ TeV. We find that the effect of adding the HL–LHC pseudo–data is to reduce the PDF uncertainties in a range of $p_T^{\mathrm{jet}}$ and $E_T^\gamma$. In the case of direct photon production, the effects are seen across most of the $E_T^\gamma$ range, while in the case of inclusive jet production they are more localised around the $p_T^{\mathrm{jet}}\sim 1$ TeV region.

**Fig. 11:**
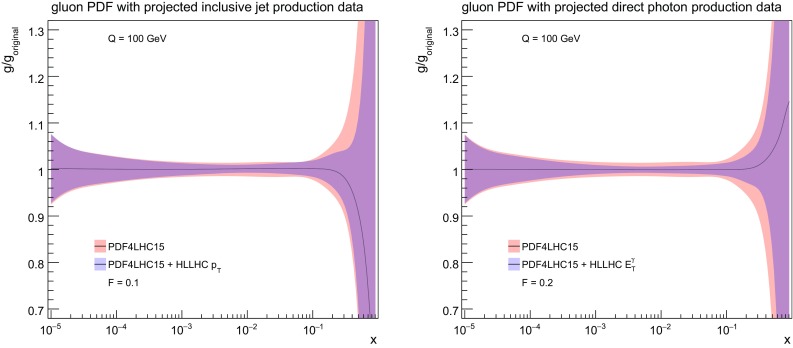
As in Fig. 6, now for the gluon PDF after including the HL–LHC pseudo–data on inclusive jet production (left plot) and on direct photon production (right plot)

Concerning the impact of these two types of HL–LHC pseudo–data on the PDFs, shown in Fig. 11, one sees that in both cases there is a visible reduction on the gluon uncertainties at both intermediate and large values of *x*, of comparable size at high *x*, while at intermediate *x* the isolated photon data is somewhat more constraining. Note again that as expected the shift in the central values of the PDFs after profiling are much smaller than the PDF uncertainties themselves. Taking into account the results found when adding top quark pair production data into the fit, see Fig. 8, a clear picture emerges showing that the HL–LHC measurements will provide particularly stringent constraints on the large–*x* gluon PDF, in addition to those on the quarks.

### *W* production in association with charm quarks

**Fig. 12:**
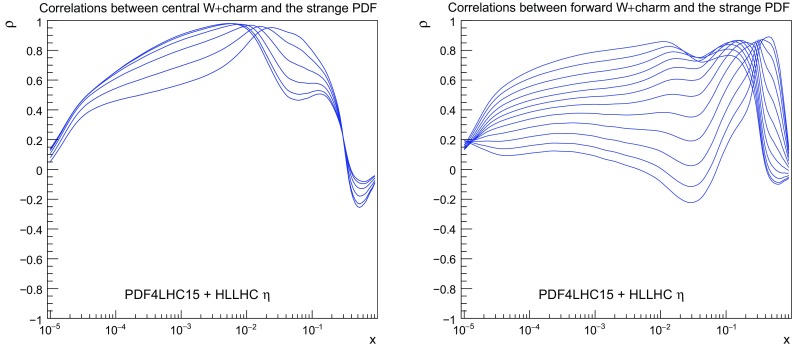
As in Fig. 4, now for the correlation coefficient between the strange PDF and the lepton rapidity distributions in *W*+charm production pseudo–data in the central rapidity region (left) and in the forward region (right plot)

We now consider the impact of *W* production in association with charm quarks, which provides direct information on the strange content of the proton. As explained in Sect. 2.1 we have generated pseudo–data both in the central rapidity region, relevant for ATLAS and CMS, and in the forward rapidity region, relevant for LHCb. In Fig. 12 we show the correlation coefficient between the strange PDF and the lepton rapidity distributions in *W*+charm production pseudo–data both for the central and the forward rapidity regions. We can see that indeed there is a large correlation between the strange PDF and the *W*+charm production pseudo–data in a broad range of *x* values. For the case of central production, we find $\rho \ge 0.9$ in the range of $10^{-3}\le x \le 0.1$, while for forward production the correlation coefficient $\rho$ peaks at a somewhat smaller value, and covers a broader range in *x*, with in particular a coverage of the small and large–*x* regions that is complementary to the central production pseudo–data.

**Fig. 13:**
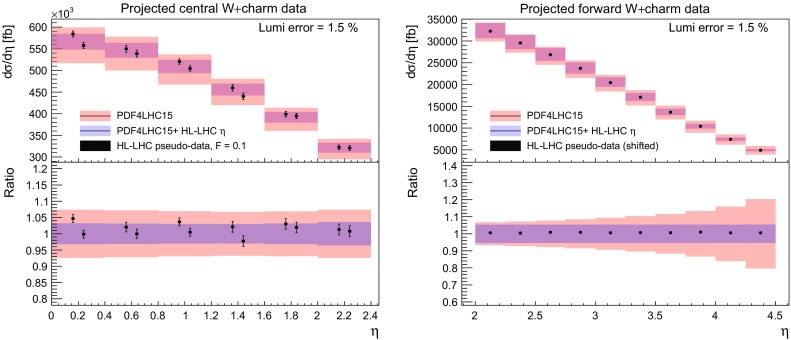
As in Fig. 5, now for *W*+charm production in the central (left plot) and forward (right plot) rapidity regions. In the right plot only the statistical errors are shown, while the data have been shifted by the dominant correlated source of uncertainty, namely the 5% normalization uncertainty

The comparison between the HL–LHC pseudo–data and the corresponding theoretical predictions for *W*+charm production both in the central and forward regions are collected in Fig. 13. In the central region, we see a clear reduction of the PDF uncertainties after including the pseudo–data into the fit, by around a factor two. This reduction of uncertainty is approximately constant as a function of the lepton rapidity. At forward rapidities instead, we find that before adding the pseudo–data the PDF uncertainties grow very fast with rapidity, reaching up to 30% for $\eta _l \simeq 4.5$, while after including it they are markedly reduced and become more or less constant with rapidity as in the central region. Taking into account the correlation coefficients shown in Fig. 12, these results indicates that *W*+charm production in the forward region provides valuable constraints on the large–*x* strangeness, which is currently affected by large uncertainties.

**Fig. 14:**
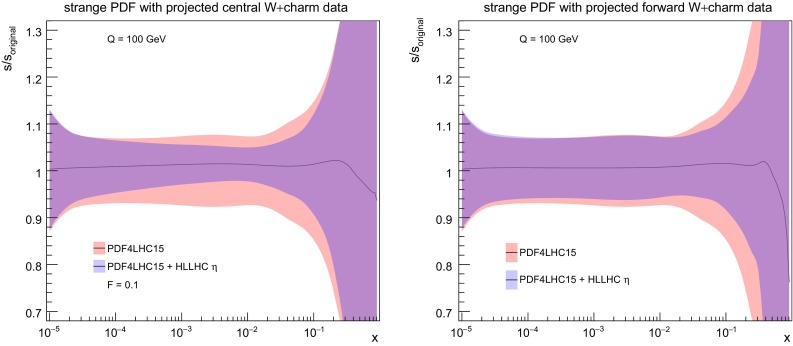
As in Fig. 6, now for the strange quark PDF including the HL–LHC pseudo–data on *W*+charm production in the central (left plot) and in the forward region (right plot)

This PDF uncertainty reduction on strangeness upon the addition of the $W+$charm pseudo–data is quantified in Fig. 14. For central production, we find that indeed most of the PDF uncertainty reduction is concentrated in the region $10^{-3}\lesssim x \lesssim 0.1$, while the large–*x* region is affected only moderately. For the pseudo–data in the forward region instead, there is a superior reduction of the PDF uncertainties in the large–*x* region. The nice complementarity seen from Fig. 14 illustrates the usefulness of combine PDF–sensitive measurements in the central rapidity region with those of the forward region.

**Fig. 15:**
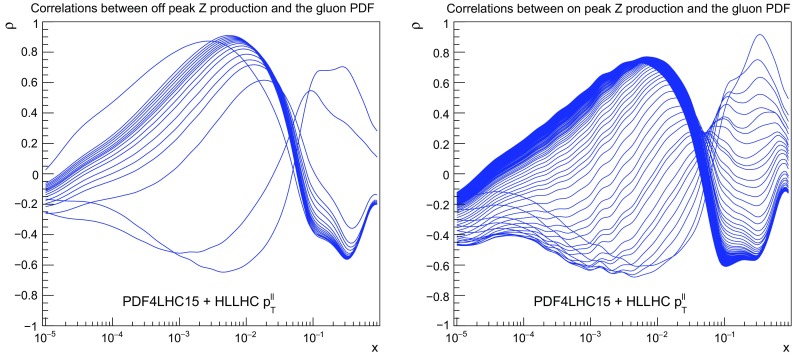
As in Fig. 4, now for the correlation coefficients between the gluon PDF and the *Z* transverse momentum distributions in the central rapidity region, for the dilepton invariant mass $10\,\mathrm{GeV}\le m_{ll}\le 20$ GeV (left plot) and $66\,\mathrm{GeV}\le m_{ll}\le 116$ GeV (right plot)

**Fig. 16:**
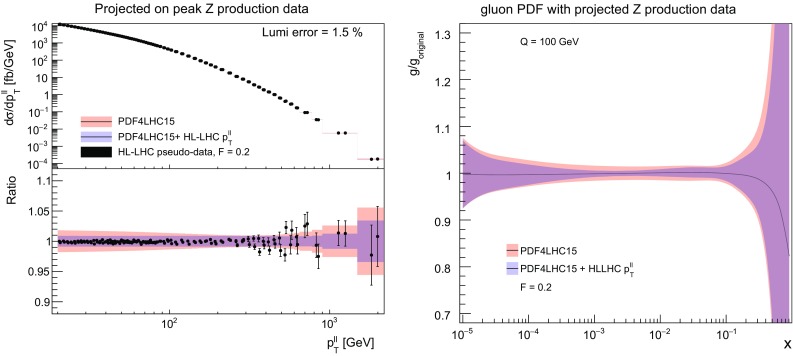
Left: as in Fig. 5, now for the $p_T$ distribution of *Z* bosons in the dilepton final state, in the on–peak bin defined by $66~\mathrm{GeV}\le m_{ll} \le 116~\mathrm{GeV}$. Right: as in Fig. 6, now for the gluon PDF after including the HL–LHC *Z* transverse momentum pseudo–data in the fit

### The transverse momentum of *Z* bosons

We complete the study of the impact of individual HL–LHC pseudo–data on the PDFs with the transverse momentum of *Z* bosons, a process which is known to influence the gluon and the quark PDFs at intermediate values of *x*. We will show the data vs. theory comparison only for the dominant dilepton invariant mass bin, $66\,\mathrm{GeV}\le m_{ll} \le 116$ GeV, although in the PDF profiling the effects of all the six $m_{ll}$ bins are being taken into account. To begin with, in Fig. 15 we show the correlation coefficients between the gluon PDF and the *Z* transverse momentum distributions in the central rapidity region, for the dilepton invariant mass $10\,\mathrm{GeV}\le m_{ll}\le 20$ GeV and $66\,\mathrm{GeV}\le m_{ll}\le 116$ GeV. As we can see from this comparison, the pseudo–data on the *Z* $p_T$ provides information on the gluon PDF, being mostly sensitive at $x\simeq 10^{-2}$, as well as around $x\simeq 0.3$ in the case of the on–peak dilepton invariant mass bin.

**Table 2:** The three scenarios for the systematic uncertainties of the HL–LHC pseudo–data that we assume in the present study. These scenarios, ranging from conservative to optimistic, differ among them in the reduction factor $f_\mathrm{red}$, Eq. (2.2), applied to the systematic errors of the reference 8 TeV or 13 TeV measurements. We also indicate in each case the name of the corresponding LHAPDF grid

Scenario	$f_\mathrm{red}$ (8 TeV)	$f_\mathrm{red}$ (13 TeV)	LHAPDF set	Comments
A	1	0.5	PDF4LHC_nnlo_hllhc_scen1	Conservative
B	0.7	0.36	PDF4LHC_nnlo_hllhc_scen2	Intermediate
C	0.4	0.2	PDF4LHC_nnlo_hllhc_scen3	Optimistic

The comparison between HL–LHC pseudo–data and theoretical predictions in the on–peak bin defined by $66~\mathrm{GeV}\le m_{ll} \le 116~\mathrm{GeV}$ is shown in Fig. 16, where we can see that coverage up to $p_T^{ll}\simeq 3$ TeV is expected, similar as in the case of direct photon production. We find a moderate reduction in the PDF uncertainties once the HL–LHC pseudo–data is added to the fit by means of Hessian profiling. Concerning its effects on the gluon, we see that the *Z* $p_T$ measurements provide valuable information in the intermediate *x* region between $10^{-3}$ and $10^{-2}$ with a clear reduction of PDF uncertainties even if in this region these were quite small to begin with.

## Ultimate PDFs with HL–LHC pseudo–data

In this section we combine the complete set of HL–LHC pseudo–data listed in Table 1 to produce the final profiled PDF sets, which quantify the impact of future HL–LHC measurements on our knowledge of the quark and gluon structure of the proton.

In Table 2 we list the three scenarios for the systematic uncertainties of the HL–LHC pseudo–data that we assume in the present analysis. These scenarios, ranging from more conservative to more optimistic, differ among them in the reduction factor $f_\mathrm{red}$, Eq. (2.2), applied to the systematic errors of the reference 8 TeV or 13 TeV measurements. In particular, in the optimistic scenario we assume a reduction of the systematic errors by a factor 2.5 (5) as compared to the reference 8 TeV (13 TeV) measurements, while for the conservative scenario we assume no reduction in systematic errors with respect to 8 TeV reference. We also indicate in each case the name of the corresponding LHAPDF grid. Reassuringly, as we show below, the qualitative results of our study depend only mildly in the specific assumption for the values of $f_\mathrm{red}$.

In this section, we study how the HL–LHC pseudo–data constraints the parton distributions and the PDF luminosities for proton–proton collisions at $\sqrt{s}=14$ TeV. Then we present an initial study with some representative implications of the ultimate PDFs for LHC phenomenology.

### Parton distributions

**Fig. 17:**
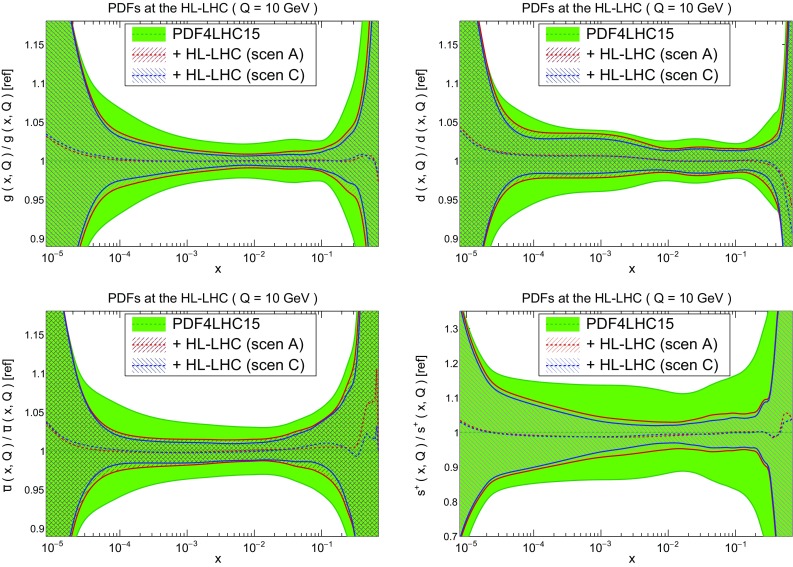
Comparison of the PDF4LHC15 set with the HL–LHC profiled set in scenarios A and C, defined in Table 2. We show the gluon, down quark, up anti–quark, and total strangeness at $Q=10$ GeV, normalized to the central value of the PDF4LHC15 baseline. The bands correspond to the one–sigma PDF uncertainties

In Fig. 17 we present a comparison of the baseline PDF4LHC15 set with the profiled sets based on HL–LHC pseudo–data from scenarios A (conservative) and C (optimistic) as defined in Table 2. Specifically, we show the gluon, down quark, up anti–quark, and total strangeness at $Q=10$ GeV, normalized to the central value of the PDF4LHC15 baseline. In this comparison, the bands correspond to the one–sigma PDF uncertainties.

First of all, we observe that the impact of the HL–LHC pseudo–data is reasonably similar in the conservative and optimistic scenarios. This is not so surprising, as we have explicitly chosen those datasets which will benefit from a significant improvement in statistics, and these tend to lie in kinematic regions where the PDFs themselves are generally less well determined, see the discussion in Sect. 2. Therefore, the dominant reason for the observed reduction of PDF uncertainties is the increased statistics and the corresponding extended kinematic reach that becomes available at the HL–LHC, rather than the specific assumptions about the systematic uncertainties. This demonstrates that our results are robust against the details of the projections of how the experimental systematic uncertainties will be reduced in the HL–LHC era.

From Fig. 17 we observe a marked reduction of the PDF uncertainties in all cases. This is particularly significant for the gluon and the sea quarks, for the reason that these are currently affected by larger uncertainties than in the case of the valence quarks. In the case of the gluon PDF, there is an improvement of uncertainties across a very broad range of *x*. This is a direct consequence of the fact that we have included several HL–LHC processes that have direct sensitivity to the gluon content of the proton, namely jet, direct photon, and top quark pair production, as well as the transverse momentum of *Z* bosons.

Another striking feature of Fig. 17 concerns the strange PDF. In this case, the PDF uncertainties are reduced by almost a factor 4, from around 15% to a few percent, in a wide region of *x*. This result highlights the importance of the *W*+charm measurements at the HL–LHC, specially those in the forward region by LHCb, see Fig. 12, which represent a unique handle on the poorly known strange content of the proton. In turn, such an improved understanding of the strange PDF will feed into a reduction of theory uncertainties in crucial HL–LHC measurements such as those of $M_W$ or $\sin ^2\theta _W$.

### Partonic luminosities

**Fig. 18:**
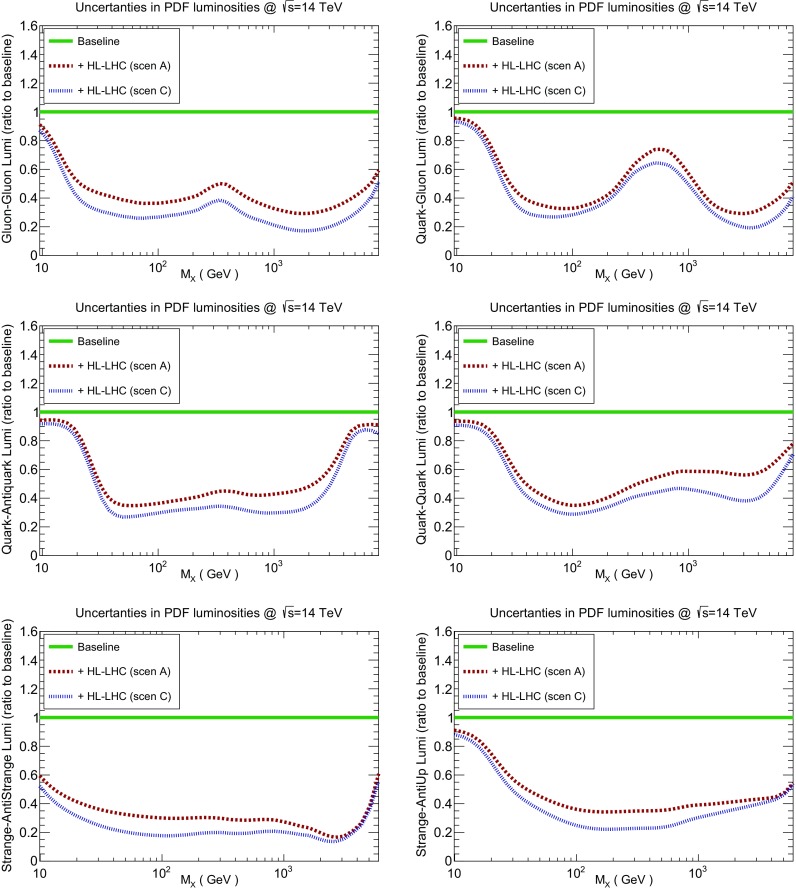
The reduction of the uncertainties in the PDF luminosities at $\sqrt{s}=14$ TeV once the HL–LHC pseudo–data has been included, compared to the PDF4LHC15 baseline. We show the *gg*, *qg*, $q\bar{q}$, *qq*, $s\bar{s}$, and $s\bar{u}$ luminosities for the conservative (A) and optimistic (C) scenarios. The average values for the PDF uncertainty reduction in different bins of $M_X$ is also reported in this figure

Next we take a look at the partonic luminosities, to quantify the improvement in the PDF uncertainties in different initial–state partonic combinations from the HL–LHC pseudo–data. In Fig. 18 we show the reduction of PDF uncertainties in the *gg*, *qg*, $q\bar{q}$, and *qq*, $s\bar{s}$, and $s\bar{u}$ luminosities at $\sqrt{s}=14$ TeV that can be expected as a consequence of adding the HL–LHC pseudo–data on top of the PDF4LHC15 baseline. Note that a value of 1 in these plots corresponds to no uncertainty reduction. As in the case of the PDF comparisons, results are shown both for the conservative (A) and optimistic (C) scenarios for our projections of the experimental systematic uncertainties.

In addition, in Table 3 we also report the average values of these PDF uncertainty reductions for three different invariant mass bins. In particular, we consider low invariant masses, $10~\mathrm{GeV}\le M_X\le 40~\mathrm{GeV}$, relevant for instance for Monte Carlo tuning and QCD studies; intermediate masses, $40~\mathrm{GeV}\le M_X\le 1~\mathrm{TeV}$, relevant for electroweak, top, and Higgs measurements; and large invariance masses, $1~\mathrm{TeV}\le M_X\le 6~\mathrm{TeV}$, relevant for searches of new heavy particles. These averages are computed from 10 points per mass bin, logarithmically spaced in $M_X$. In Table 3, the values shown outside (inside) the brackets correspond to the optimistic (conservative) scenario.

**Table 3:** The reduction of the PDF uncertainties compared to the PDF4LHC15 baseline for different initial partonic combinations (that is, a value of 1 corresponds to no reduction at all). Results are presented for three different bins of the invariant mass $M_X$ of the produced system, averaging over 10 points logarithmically spaced within each bin. The values shown outside (inside) the brackets correspond to the optimistic (conservative) scenario. The corresponding results differential in $M_X$ are presented in Fig. 18

Ratio to baseline	$10~\mathrm{GeV}\le M_X\le 40~\mathrm{GeV}$	$40~\mathrm{GeV}\le M_X\le 1~\mathrm{TeV}$	$1~\mathrm{TeV}\le M_X\le 6~\mathrm{TeV}$
gluon–gluon	0.50 (0.60)	0.28 (0.40)	0.22 (0.34)
gluon–quark	0.66 (0.72)	0.42 (0.45)	0.28 (0.37)
quark–quark	0.74 (0.79)	0.37 (0.46)	0.43 (0.59)
quark–antiquark	0.71 (0.76)	0.31 (0.40)	0.50 (0.60)
strange–antistrange	0.34 (0.44)	0.19 (0.30)	0.23 (0.27)
strange–antiup	0.67 (0.73)	0.27 (0.38)	0.38 (0.43)

From the comparisons in Fig. 18 and Table 3, we observe again that the reduction in the uncertainties of the PDF luminosities is rather robust with respect to the assumed projections for the experimental systematic uncertainties. For instance, for intermediate values of the final–state invariant mass, $40~\mathrm{GeV}\le M_X\le 1~\mathrm{TeV}$, we find that for all the partonic initial states the reduction factor varies between 0.28 and 0.40 (0.42 and 0.45, 0.31 and 0.40) in the optimistic and conservative scenario for the gluon–gluon (gluon–quark, quark–antiquark) luminosities. These results again reinforce our conclusion that the results of this study are only mildly sensitive to the details of the projected pseudo–data.

We find that in the intermediate $M_X$ bin the reduction of PDF uncertainties ranges approximately between a factor 2 and a factor 5, depending on the specific partonic channel and the scenario for the systematic errors. For example, for the gluon–gluon luminosity in the range relevant for Higgs production in gluon fusion, one finds a reduction by almost a factor 4 in the optimistic scenario. The improvement in the strange–initiated processes is also remarkable, for example the PDF uncertainties in the $s\bar{s}$ luminosity are expected to be reduced by a factor 5 (3) in the optimistic (conservative) scenario. Recall that strange–initiated processes are important for a variety of LHC analysis, from measurements of $M_W$ and $\sin ^2\theta _W$ to searches for BSM $W'$ bosons. We also find that the uncertainties in quark–antiquark luminosities, relevant for example for precision electroweak measurements, are expected to be reduced by up to a factor 3 in this invariant mass range.

Similar improvements in the PDF luminosities are found in the high mass region, $M_X\ge 1$ TeV, directly relevant for BSM searches. For instance, in the optimistic scenario, the PDF error reduction at higher masses is expected to be as large as a factor 5 for the gluon–gluon luminosity. Again this is a consequence of the inclusion in the profiling of gluon–dominated processes such as $t\bar{t}$ and inclusive jets that at the HL–LHC, which cover the region up to 6 TeV, see Fig. 2. The impact of the HL–LHC pseudo–data is less marked for the quark–quark and quark–antiquark luminosities in this high–mass region, due to the fact that of the data points included in the profiling only a fraction of them are both quark–initiated and cover the large–*x* region.

It is worth emphasizing again here that the list of processes studied in this work and summarised in Table 3 are just a subset of those HL–LHC measurements with PDF–constraining potential. Therefore, it is conceivable that the actual reduction of PDF errors presented in Table 3 would actually be more significant than our estimates here.

### Implications for HL–LHC phenomenology

We now turn to present some representative results of the phenomenological implications that these “ultimate” PDFs will have at the HL–LHC, both for processes within the SM and beyond it. It is beyond the scope of this work to carry out a comprehensive phenomenological study, and we refer the reader to the upcoming Yellow Report [27] describing the physics case of the HL–LHC, where more detailed projections and analyses will be presented.

Let us begin by assessing the PDF impact of HL–LHC measurements on representative Standard Model processes. In particular, we consider diphoton production, dijet production, and Higgs production in gluon fusion, both inclusive and in association with a hard jet. In the following all cross sections have been computed at $\sqrt{s}=14$ TeV using leading order (LO) matrix elements with MCFMv8.2 [58] and applying the standard ATLAS/CMS central acceptance cuts. Since the comparison is restricted to ratios of cross sections, the LO calculation is sufficient to illustrate the impact of the improvement in the PDF uncertainties in each of these processes. Indeed, we are only interested here in illustrating the relative impact of the PDF error reduction, rather than providing state–of–the–art predictions for the rates, which will be presented elsewhere in the Yellow Report [27].

**Fig. 19:**
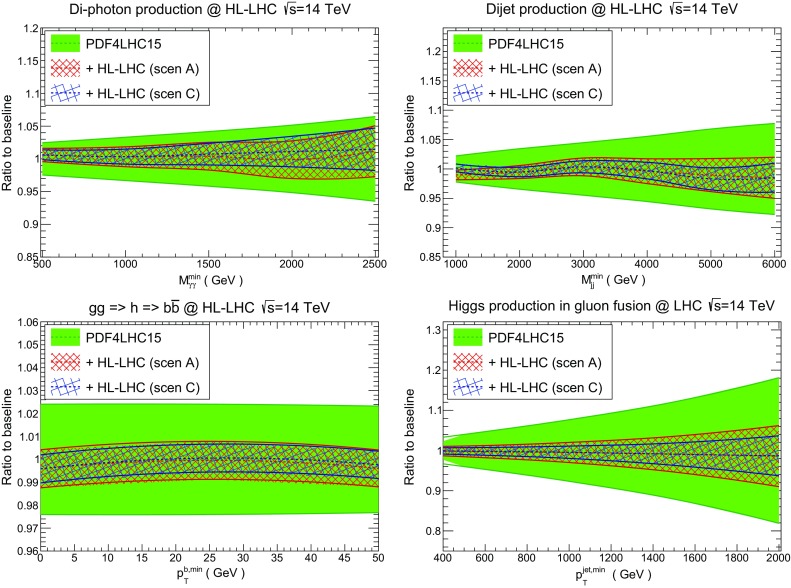
Comparison of the predictions for representative SM cross sections at $\sqrt{s}=14$ TeV between the PDF4LHC15 baseline and the HL–LHC profiled sets in the conservative (A) and optimistic (C) scenarios. Results are shown normalised to the central value of PDF4LHC15. The upper plots show the diphoton (left) and dijet (right plot) production cross sections as a function of the minimum invariant masses of the final state, $M_{\gamma \gamma }^{\mathrm{min}}$ and $M_{jj}^{\mathrm{min}}$ respectively. The bottom plots show Higgs boson production in gluon fusion with heavy top quark effective theory, both inclusive and decaying into $b\bar{b}$ as a function of $p_T^{b,\mathrm min}$ (left), and then in association with a hard jet as a function its transverse momentum $p_\mathrm{T}^{jet,\mathrm min}$ (right plot). The calculations have been performed using MCFM8.2 with leading–order matrix elements

First of all, we show the production cross sections of pairs of photons (left) and of jets (right) in the upper panels of Fig. 19. We compare the PDF4LHC15 baseline with the HL–LHC profiled PDF sets in the conservative (A) and optimistic (C) scenarios of Table 2, normalised to the central value of PDF4LHC15. In the considered kinematic regions, these two processes are mostly sensitive to the quark–antiquark initial state, and to the quark–gluon and quark–(anti)quark initial states, respectively. The cross sections are presented as a function of the minimum invariant mass of the final state, $M_{\gamma \gamma }^{\mathrm{min}}$ and $M_{jj}^{\mathrm{min}}$ respectively, in order to facilitate their comparison with the corresponding PDF luminosities shown in Fig. 18.

From this comparison, we find again that both the optimistic and conservative scenarios lead to similar results in terms of the expected reduction of the PDF uncertainties in the entire kinematical range accessible at the HL–LHC. In the case of dijet production, we find that PDF uncertainties could be reduced down to $\simeq 2\%$ even for invariant masses as large as $M_{jj}=6$ TeV. The resulting improved theory predictions for dijet production should also become relevant at the HL–LHC for BSM searches, *e.g.* for quark compositeness [73, 74]. Note that since the initial partonic states are the same, similar improvements are expected for inclusive jet production, see also Fig. 10, as well as for multijet production. Similar considerations apply for diphoton production, where the expected PDF error reduction is a bit less marked since it is driven by the quark–antiquark luminosity, see also the comparisons in Table 3.

**Fig. 20:**
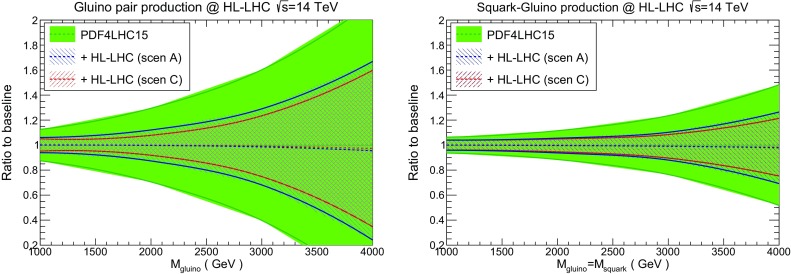
The cross sections for high–mass supersymmetric particle production at $\sqrt{s}=14$ TeV, comparing the predictions of the PDF4LHC15 baseline with those of the HL–LHC PDF sets in the conservative (A) and optimistic (C) scenarios, normalised to the central value of PDF4LHC15. We show the results corresponding to gluino pair production (left) and squark–gluino production (right). The cross sections have been evaluated with Pythia8.235 using leading–order matrix elements and the SLHA2 benchmark point as model input

In the two lower plots of Fig. 19, we present the corresponding comparisons for the case of Higgs boson production via gluon fusion, using heavy top quark effective theory. In the case of inclusive production with decay into bottom quarks (left plot), we find that the constraints from HL–LHC measurements are expected to reduce PDF uncertainties down to the $1\%$ level. Needless to say, this will directly benefit the characterisation of the Higgs sector at the HL–LHC, where a few percent is the typical uncertainty target for the determination of its couplings. In the case of Higgs boson production in association with a hard jet (right plot), also there we find a marked error reduction, indicating that PDF uncertainties in the Higgs transverse momentum distribution could be reduced down to the $\simeq$2% level in the entire kinematical range relevant at the HL–LHC. We recall that the large Higgs transverse momentum region is sensitive to new heavy particles running in the loops as well as to BSM effects such as partial Higgs compositeness [75].

As we have discussed above in Sects. 4.1 and 4.2, the impact of the HL–LHC pseudo–data is also significant in the large–*x* region, which in turn corresponds to large invariant masses for the PDF luminosities. This is of course an important region for the searches of BSM heavy particles, where PDF uncertainties often represent the dominant source of theoretical uncertainty. With this motivation, to illustrate the benefits that HL–LHC measurements will provide for BSM searches we consider here high–mass supersymmetric (SUSY) particle production at $\sqrt{s}=14$ TeV, where the HL–LHC reach extends to sparticles masses up to around $M\simeq 3$ TeV. While we use SUSY production as a benchmark process, our results also apply to the production of other heavy particles predicted in different BSM scenarios.

In Fig. 20 we show the comparison between the PDF4LHC15 predictions with the corresponding results from the profiled PDF sets with HL–LHC pseudo–data, normalised to the central value of the PDF4LHC15 baseline. As in Fig. 19, we provide results for scenarios A and C, the conservative and optimistic ones respectively. Specifically, we show the cross sections for gluino–gluino and squark–gluino production at $\sqrt{s}=14$ TeV – similar conclusions are derived from squark–squark and squark–antisquark production. The theoretical calculations have been obtained using leading order (LO) matrix elements with Pythia8.235 [76] and assuming the SLHA2 benchmark point [77], for a range of sparticle masses within the HL–LHC reach. For simplicity, underlying event and multiple interactions have been switched off in the calculation. Again, we are not interested here in providing state–of–the–art predictions for the event rates, which can be found elsewhere [78].

From the comparisons in Fig. 20, we can see that the constraints on the PDFs expected from the HL–LHC data permit a significant reduction of the uncertainties in the high–mass SUSY cross sections. The size of this reduction is consistent with the corresponding results at the level of luminosities, reported in Fig. 18 and Table 3, recalling that gluino–gluino and gluino–squark production are driven by the gluon–gluon and gluon–quark initial states respectively [5]. For instance, for gluino pair–production with $M_{\widetilde{g}}=3$ TeV, the PDF uncertainties are reduced from $\simeq 60\%$ to $\simeq 20\%$ in the optimistic scenario. A somewhat milder reduction is found for the squark–gluino cross sections. For squark–squark and squark–antisquark production, driven by the quark–quark and quark–antiquark initial states respectively, a PDF uncertainty reduction by around a factor two at high masses is found, consistently with Table 3.

To summarise, the initial phenomenological study presented in this section nicely illustrates the internal coherence of the HL–LHC physics program: high precision SM measurements will lead to a much improved understanding of the quark and gluon structure of protons, which in turn will benefit many other important analyses, from the characterisation of the Higgs sector to the searches of new heavy particles.

## Summary

In this study, we have quantified the expected constraints that precision HL–LHC measurements will impose on the quark and gluon structure of the proton. To achieve this goal, we have assessed the impact of a range of relevant PDF–sensitive processes, from weak gauge boson and jet production to top quark and photon production. Moreover, we have studied the robustness of our results with respect to different projections for the experimental systematic uncertainties, from a more conservative one, where systematics are assumed to have the same size as in current measurements, to a more optimistic one, where they are markedly reduced.

Our main finding is that HL–LHC data has the potential to significantly reduce the PDF uncertainties in a wide kinematic range and for all relevant partonic final states. This is true both for the region of intermediate invariant masses, relevant for precision Higgs, electroweak, and top quark measurements, as well as in the TeV region relevant for searches of new heavy particles. Even in the most conservative scenario, in the region $M_X\gtrsim 40$ GeV we find that HL–LHC measurements can reduce PDF uncertainties by at least a factor between 2 and 3 as compared to the current PDF4LHC15 baseline. The PDF constraining information from the HL–LHC is expected to be specially significant for gluon– and for strange–initiated processes. We also find that the quark–antiquark luminosity at the electroweak scale, a central input for legacy LHC measurements such as $M_W$ and $\sin ^2\theta _W$, could be improved by more than a factor 3 in the optimistic scenario.

This improved knowledge of the quark and gluon structure of the proton which will become possible at the HL–LHC will directly benefit a number of phenomenologically important process, due to the reduction of the associated theoretical errors. For instance, the PDF uncertainties in Higgs production in gluon fusion can be reduced down to $\lesssim 2\%$ for the entire range of Higgs transverse momenta accessible at the HL–LHC. Likewise, PDF uncertainties in high–mass supersymmetric particle production can be decreased by up to a factor 3, with a similar impact expected for other BSM scenarios. This improvement should strengthen the bounds derived in the case of null searches, or facilitate their characterisation in the case of an eventual discovery. Similar improvements are found for Standard Model process, for example dijet production, which provides a unique opportunity to measurement the running of the strong coupling constant at the TeV scale. More detailed studies of the phenomenological implications of our study will be presented in the upcoming HL–LHC Yellow Report. Two caveats are relevant at this point. First, it should be emphasised again that in this study we have only considered a subset of all possible measurements of relevance for PDF fits. There are certainly processes for which data is and will be available, such as multijet production and single top production, that we have not considered here. Moreover, we can also reasonably expect that various new processes may be added to the PDF toolbox on the rather long timescales we consider here. Thus, we may certainly expect further constraints to become available for PDF studies by the end of HL–LHC running.

Second, in this study we have ignored any possible issues such as data incompatibilities, limitations of the theoretical calculations, or issues affecting the data correlation models. These are common in PDF fits, and indeed have already been found when comparing theory calculations against existing LHC data from Runs I and II. Such potential problems may eventually limit the PDF constraining power, in comparison to the estimates presented in this work, when the actual global fit with real HL–LHC data is performed. Clearly, such questions can only be tackled once the HL–LHC measurements are carried out, and indeed doing so will present an important programme of experimental and theoretical PDF–related work on its own. We cannot anticipate such work in our present study, which instead represents our best quantitative projections using our current knowledge. The results of this study are made publicly available in the LHAPDF6 format [46], with the grid names listed in Table 2 for the three scenarios that have been considered. These three grid files can be downloaded from:

https://data.nnpdf.science/HLLHC_YR/PDF4LHC15_nnlo_hllhc_scen1.tgz

https://data.nnpdf.science/HLLHC_YR/PDF4LHC15_nnlo_hllhc_scen2.tgz

https://data.nnpdf.science/HLLHC_YR/PDF4LHC15_nnlo_hllhc_scen3.tgz

The “ultimate” PDFs produced in this exercise can then be straightforwardly applied to other physics projections of HL–LHC processes, taking into account our improved knowledge of the partonic structure of the proton which is expected by then. We believe that the results of this work represent an important ingredient towards sharpening as much as possible the physics reach of the LHC in its upcoming high–luminosity era.

## Footnotes

1 See also [28] for a recent review of the potential of the LHCb experiment in the HL–LHC era.

2 Note that sometimes in this paper we will for brevity use the shorthand ‘fit’, but it always understood that a profiling has been performed rather than a full refit.
